# Magnetic resonance imaging aspects after surgical repair of knee cartilage: pictorial essay

**DOI:** 10.1590/0100-3984.2019.0020

**Published:** 2020

**Authors:** Anthony Reis Mello Souza, Adham do Amaral e Castro, Eduardo Kaiser Ururahy Nunes Fonseca, Letícia Maria Araújo Oliveira Nunes, Eduardo Baptista, Luiz Guilherme de Carvalho Hartmann

**Affiliations:** 1 Departamento de Imagem - Hospital Israelita Albert Einstein, São Paulo, SP, Brazil.

**Keywords:** Knee, Cartilage, Magnetic resonance imaging, Joelho, Cartilagem, Ressonância magnética

## Abstract

Radiologists should be familiar with the main techniques of knee cartilage repair and the imaging methods available for its evaluation, in order to optimize the postoperative follow-up of patients. The objective of this study was to present a series of clinical cases seen at our facility, illustrating the main techniques necessary for the repair of knee cartilage, as well as the magnetic resonance imaging techniques used in the postoperative evaluation and the relevant radiological findings.

## Abstract (pt)

Radiologistas devem estar familiarizados com as principais técnicas de reparo cirúrgico da cartilagem do joelho e os métodos de imagem disponíveis para sua avaliação, no intuito de otimizar o seguimento pós-operatório do paciente. O presente estudo tem por finalidade, por meio de uma coletânea de casos do nosso serviço, ilustrar as principais técnicas cirúrgicas aplicadas ao reparo da cartilagem do joelho, bem como as técnicas de ressonância magnética usualmente utilizadas na avaliação pós-operatória e os achados radiológicos relevantes.

## INTRODUCTION

Cartilaginous tissue lines the various joints of the body, having the basic function of absorbing and better distributing the loads applied. Its basic properties are plasticity and lubrication. It is rich in type II collagen fibers and is divided into four distinct layers of chondrocytes^(1)^.

The healing potential of articular cartilage is quite limited because, unlike most tissues in the body, it is avascular and therefore has a poor inflammatory/repair response to injury. To overcome that biological limitation, several studies over the years have targeted the knee cartilage in attempts to develop surgical techniques to stimulate healing, restore integrity, or even regenerate tissue^(1,2)^.

Studies have shown that the cartilage defect is filled by fibrocartilage (rich in type I collagen fibers), which has biomechanical properties different from those of the normal hyaline cartilage. In order to achieve a favorable postoperative evolution, patients undergoing surgery to repair cartilage in the knee should be monitored, clinically and radiologically, to identify cases of treatment failure as early as possible^(2)^.

In this pictorial essay, we had multiple objectives. We attempted to illustrate the main surgical techniques for knee cartilage repair. We also identify the magnetic resonance imaging (MRI) sequences typically used in the postoperative evaluation and the relevant imaging findings, as well as demonstrating complementary diagnostic imaging techniques (T2 mapping).

We have reviewed and illustrated cases of patients treated with a number of different surgical techniques for knee cartilage repair. The techniques addressed are microfracture, mosaicplasty, repair by stem-cell regeneration, surgical fixation of a chondral fragment, and the use of biomembranes^(1-6)^.

## INITIAL CONCEPTS

The main objectives of surgical repair of patellofemoral cartilage are to reduce symptoms, to promote cartilage healing, and to prevent or delay the onset of osteoarthritis. Surgical repair of patellofemoral cartilage damage is especially important for professional athletes^(1)^.

Arthroscopy continues to be the gold standard for the evaluation of cartilage damage and for the surgical repair of its defects. However, it is an invasive procedure, intrinsically associated with morbidity, and is usually reserved for treatment after evaluation by diagnostic imaging. In this context, MRI plays a fundamental role in the preoperative and postoperative evaluation^(1,2)^.

Two-dimensional fast spin-echo or turbo spin-echo MRI sequences provide excellent tissue contrast and faster acquisition times. Because fast spin-echo is the type of acquisition most commonly used for the clinical evaluation of cartilage lesions, it is part of the cartilage imaging protocol recommended by the International Cartilage Repair Society^(1,11)^. The most common MRI acquisitions for morphological evaluation of cartilage include proton density-weighted sequences, sequences with intermediate echo times (TEs), and T2-weighted sequences with or without fat suppression^(1,11)^.

Sequences with an intermediate TE combine the advantage of proton density contrast with T2 weighting, using a TE of 33-60 ms. Thus, it is possible to obtain signal intensity in cartilage greater than that obtained with standard T2-weighted sequences, with consequent better differentiation between cartilage and subchondral bone and less susceptibility to the effects of the magic angle phenomenon^(1,11)^.

Although T2-weighted sequences without fat suppression offer excellent contrast between cartilaginous surfaces and synovial fluid, the evaluation of cartilage is impaired due to the low signal intensity of the cartilage and the low contrast between cartilage and subchondral bone. Fat suppression techniques provide greater contrast at the interface between cartilage and subchondral bone^(1,11)^.

## CLASSIFICATION OF CHONDRAL LESIONS

A number of classification systems have been proposed for chondral lesions. In essence, the parameters evaluated (depth of the lesion and involvement of the subchondral bone) are similar among them. Therefore, in practice, we limit our radiology reports to the description of the injury, without mentioning any classification.

### Modified Outerbridge classification

**Figure 1:**
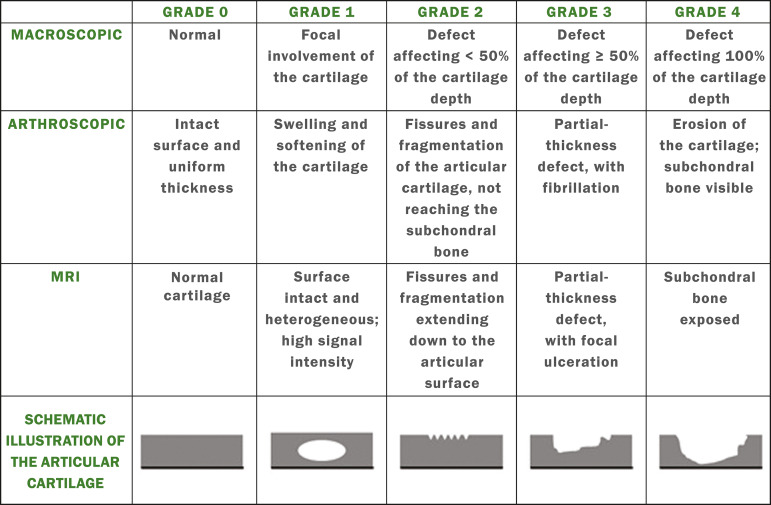
Modified Outerbridge classification.

The modified Outerbridge classification proposes to evaluate patellofemoral chondromalacia on the basis of its MRI aspects, together with its macroscopic and arthroscopic aspects^(7,8)^. The modified Outerbridge classification divides cartilage into five grades, from grade 0 to grade IV, as illustrated in Figure 1.

### Modified Noyes classification

The modified Noyes classification divides cartilage into four grades on the basis of its MRI aspects^(12)^: grade 0, normal; grade 1, increased signal intensity on T2-weighted images; grade 2a, a superficial partial-thickness chondral defect extending down to < 50% of the cartilage depth; grade 2b, a deep partial-thickness chondral defect extending down to > 50% of the cartilage depth; and grade 3, a chondral defect extending down to the calcified layer.

### International Cartilage Repair Society classification

The International Cartilage Repair Society classification divides cartilage into five overall grades, with subdivisions^(13)^. Normal cartilage is classified as grade 0. Nearly normal cartilage is classified as grade 1A (superficial lesion with soft indentation) or grade 1B (superficial lesion with fissures and cracks, with or without soft indentation). Abnormal cartilage is classified as grade 2 (lesion extending down to < 50% of the cartilage depth). Severely abnormal cartilage is classified as grade 3A (lesion extending down to > 50% of the cartilage depth), grade 3B (lesion extending down to the calcified layer), grade 3C (lesions extending down to but not through the subchondral bone), or grade 3D (same as grade 3C, plus blisters). Extremely severe cartilage abnormality is classified as grade 4A (penetration of the subchondral bone, although with a diameter smaller than that of the defect) or grade 4B (penetration of the subchondral bone with a diameter equal to that of the defect).

## SURGICAL TECHNIQUES FOR PATELLOFEMORAL CARTILAGE REPAIR

**Figure 2:**
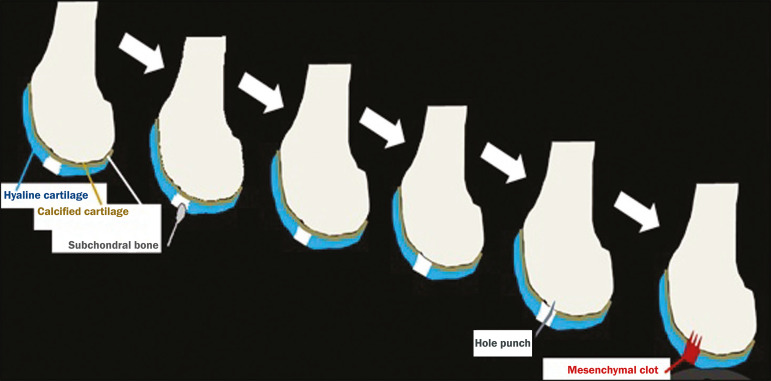
Illustration of the microfracture steps. Debridement to create a stable margin in the cartilage; curettage of the calcified cartilage layer; penetration of the subchondral bone with a hole punch to produce communications with the bone marrow; the resulting clot contains mesenchymal stem cells, which treat the defect by forming fibrocartilage.

**Figure 3:**
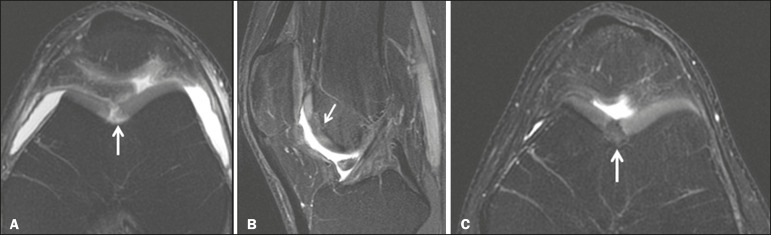
A 26-year-old man submitted to microfracture. T2-weighted fat-saturated MRI sequences. Preoperative axial image (**A**) showing a deep chondral lesion in the center of the femoral trochlea (arrow). Follow-up imaging at seven months of the procedure: sagittal and axial images (**B** and **C**, respectively), showing the microfracture trajectories, together with subchondral edema (arrows) and fibrocartilaginous tissue filling the chondral lesion site.

**Figure 4:**
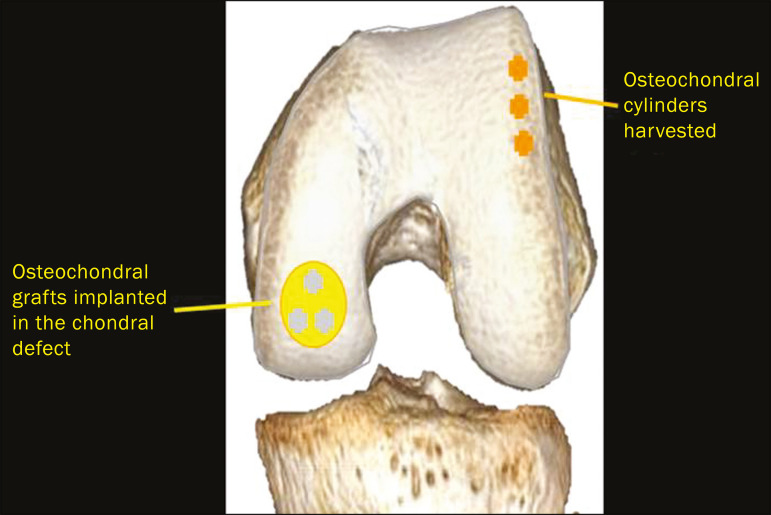
The mosaicplasty technique. The first step is debridement of the damaged cartilage, in order to stabilize the margins. Osteochondral cylinders are collected from the non–weight-bearing regions of the knee. At the site of the cartilage defect, cylindrical containers are created to receive the osteochondral grafts.

**Figure 5:**
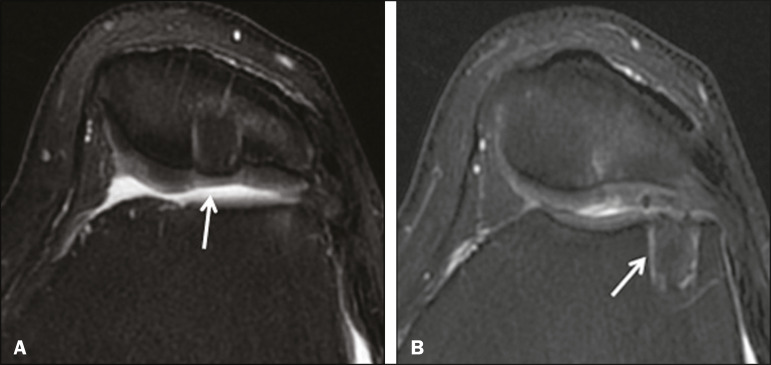
A 39-year-old man who underwent arthroscopic mosaicplasty on the lateral aspect of the patella, with a favorable evolution. Axial T2-weighted fat-saturated MRI sequences, obtained one year after the procedure, demonstrate a nearly completely filled defect, with only a small central incongruity on the articular surface (arrow in A) and minimal osteochondral irregularity at the graft collection site on the lateral aspect of the femoral trochlea (arrow in **B**).

**Figure 6:**
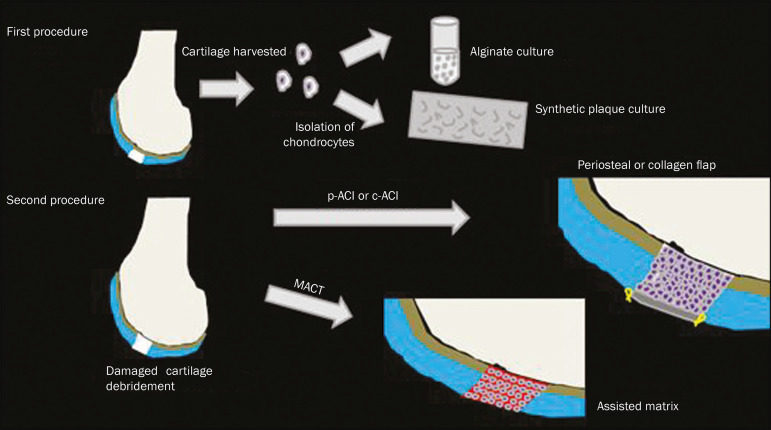
Repair by stem-cell regeneration. The first surgical procedure is arthroscopic. Cartilage is harvested from the non–weightbearing area. The tissue is prepared, and the released chondrocytes are expanded. This process can occur in two ways: in a 3D alginate culture or on a synthetic plate, for matrix-assisted autologous chondrocyte transplantation (MACT). The second procedure is open surgery. Initially, the damaged cartilage is debrided. Cultured chondrocytes can be implanted as a high-density suspension of expanded chondrocytes under a periosteal or collagen flap. There is another technique in which chondrocytes are released into the defect from the cultured plate. There is no need for sutures. c-ACI, collagen-autologous chondrocyte implantation; p-ACI, periosteal-autologous chondrocyte implantation.

**Figure 7:**
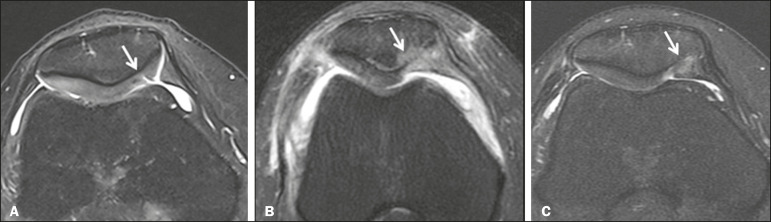
A 25-year-old woman submitted to arthroscopic injection of autologous mesenchymal stem cells into the medial aspect of the patella. Sagittal T2- weighted fat-saturated MRI sequences. Preoperative sequence (A) showing the lesion, with deep chondral fissures (arrows). Sequence obtained one week after the procedure (B), showing bone and soft-tissue edema adjacent to the repair zone (arrows), with mild joint effusion. Sequence obtained four months later (C), showing slightly irregular fibrocartilaginous tissue, with deep fissures, in the repair zone (arrows), without subchondral edema.

**Figure 8:**
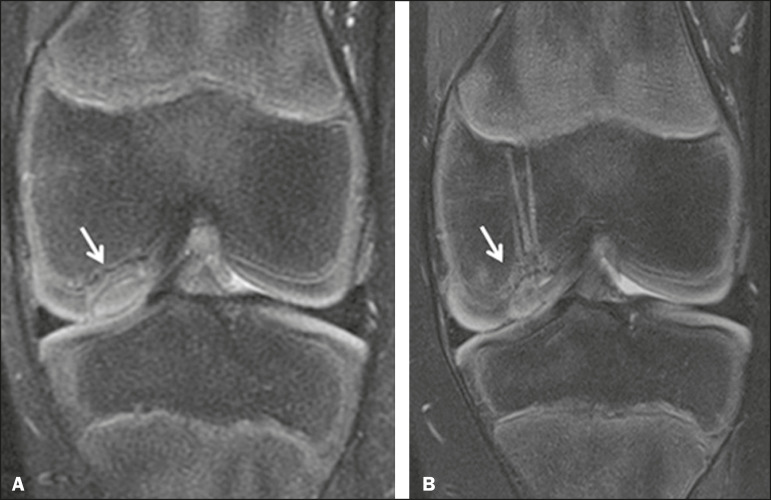
A 10-year-old boy submitted to surgical fixation of the chondral fragment. Coronal T2-weighted fat-saturated MRI sequences. Preoperative sequence (**A**) showing osteochondral involvement accompanied by bone marrow edema, without detachments in the central part of the weight-bearing area of the medial femoral condyle. Sequence obtained eight months after the procedure (**B**), showing a linear area of high signal intensity (arrow) at the subchondral cartilage/ bone interface in the inner portion of the lesion focus, representing granulation tissue. A linear area of low signal intensity at the most anterior margin of the cartilage in the focus of osteochondral injury, the site of a previous fissure, inferring coaptation, possibly with fibrocartilage tissue.

**Figure 9:**
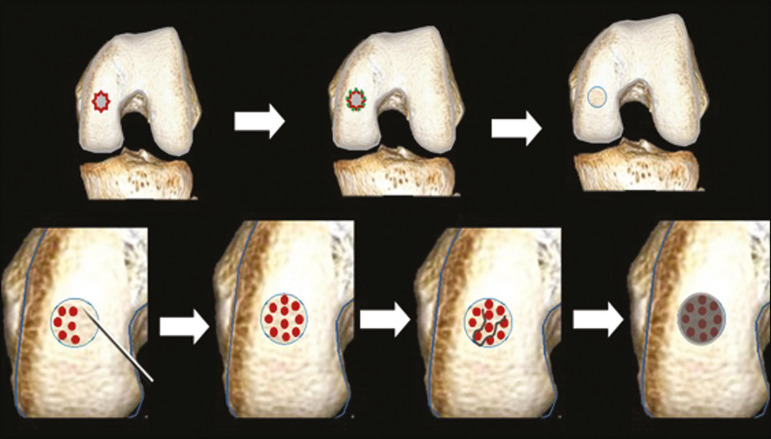
The biomembrane procedure. In the first stage of the arthroscopic procedure, the unstable and damaged cartilage is removed until regular margins are achieved. An exact impression of the defect is made using sterile aluminum foil. The model is removed and placed into the biomembrane, which is then cut out to match. In the second stage of the arthroscopic procedure, the subchondral bone at the base of the lesion is perforated from the periphery of the lesion toward the center. Fibrin glue is applied directly to the subchondral bone surrounding the perforations. The membrane, already cut to the size and shape of the lesion, is then placed in the defect, its porous face in contact with the bone surface.

**Figure 10:**
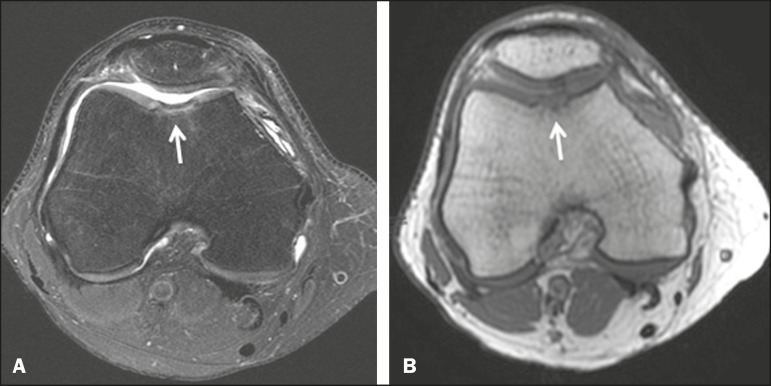
A 31-year-old man submitted to the biomembrane procedure. Postoperative axial T2-weighted fat-saturated MRI sequence (**A**) showing patellar chondropathy characterized by sparse foci of altered signal intensity, slight narrowing with superficial irregularities in the medial aspect of the vertex and deep fissure in its middle third, accompanied by mild subchondral bone edema. Signs of chondroplasty in the sulcus (arrow) and beginning of the aspects of the femoral trochlea, with a thin collagen membrane and narrow, irregular fibrocartilaginous tissue covering the subchondral bone, which is also irregular, with foci of edema. A T1-weighted sequence acquired at two months after the procedure (**B**), showing the area where the membrane joins the adjacent cartilage, where there is a slight change in signal intensity (arrow).

The main surgical techniques for patellofemoral cartilage repair are illustrated below, with a series of cases. Figures 2 and 3 depict microfracture (bone marrow stimulation); Figures 4 and 5 illustrate mosaicplasty (osteochondral graft); Figures 6 and 7 show regeneration using stem cells; Figure 8 depicts surgical fixation of a chondral fragment; and Figures 9 and 10 illustrate the use of biomembranes.

In the postoperative imaging, the following should be assessed^(11)^: the degree of repair and filling of the defect in relation to the adjacent cartilage; integration of the transplant with the adjacent cartilage and with the subchondral bone; the surface of the area repaired; the constitution and signal intensity of the repaired chondral tissue in comparison with those of the adjacent normal cartilage; the integrity of the subchondral bone (looking for signs of edema, cysts, and granulation tissue); the presence of flat osteophytes; and the presence of joint effusion.

## COMPLEMENTARY DIAGNOSTIC IMAGING TECHNIQUES

### T2 mapping

As a complementary diagnostic imaging technique, T2 mapping allows the detection of biochemical and microstructural changes in the extracellular cartilaginous matrix, even before gross morphological changes occur. It can complement MRI in defining the biomechanical quality of the cartilage repair^(9)^. Although there are more data in the literature about T2 mapping in degenerative chondral alteration treated with nonsurgical methods, various studies have validated the method for the postoperative evaluation of knee cartilage^(9-11)^.

Among the other advantages of T2 mapping are the fact that it does not require the administration of intravenous contrast injection and its easy implementation in MRI scanners. Its disadvantages include longer acquisition times for spin-echo/multi-echo sequences and its inability to assess calcified cartilage at the osteochondral junction^(9-11)^.

**Figure 11:**
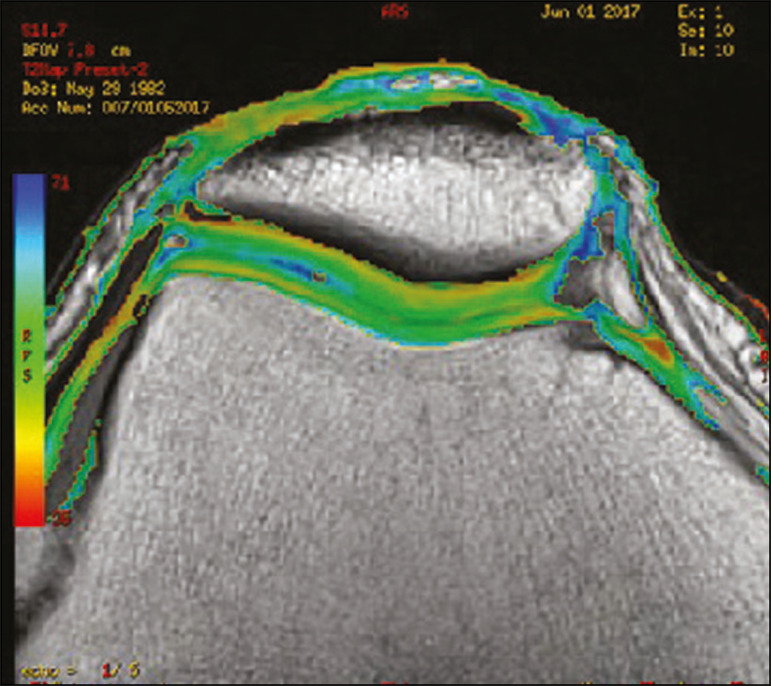
Axial MRI T2 mapping showing normal stratification of the patellofemoral cartilage surface.

**Figure 12:**
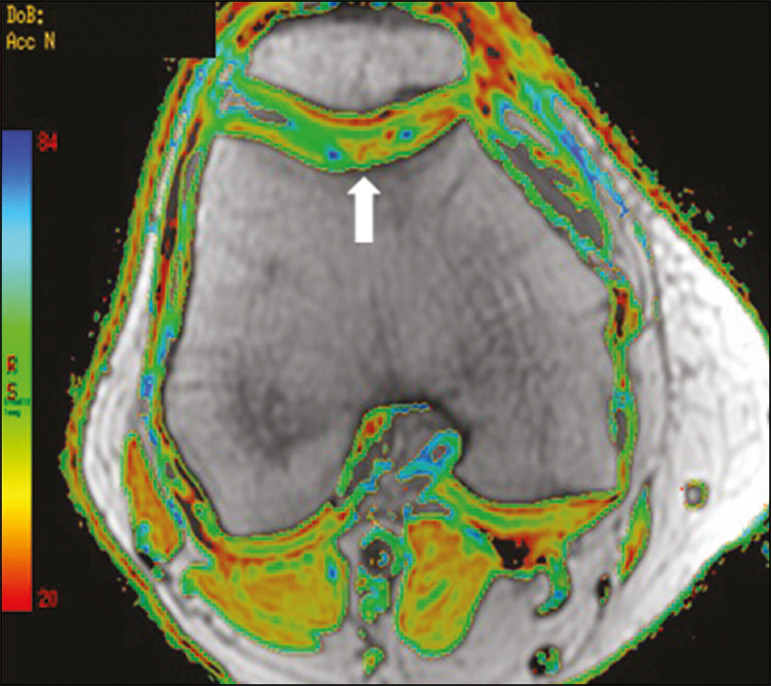
Axial T2 mapping showing stratification of the cartilage in the region where chondral manipulation was performed, similar to the hyaline cartilage of the adjacent healthy femoral trochlea.

**Figure 13:**
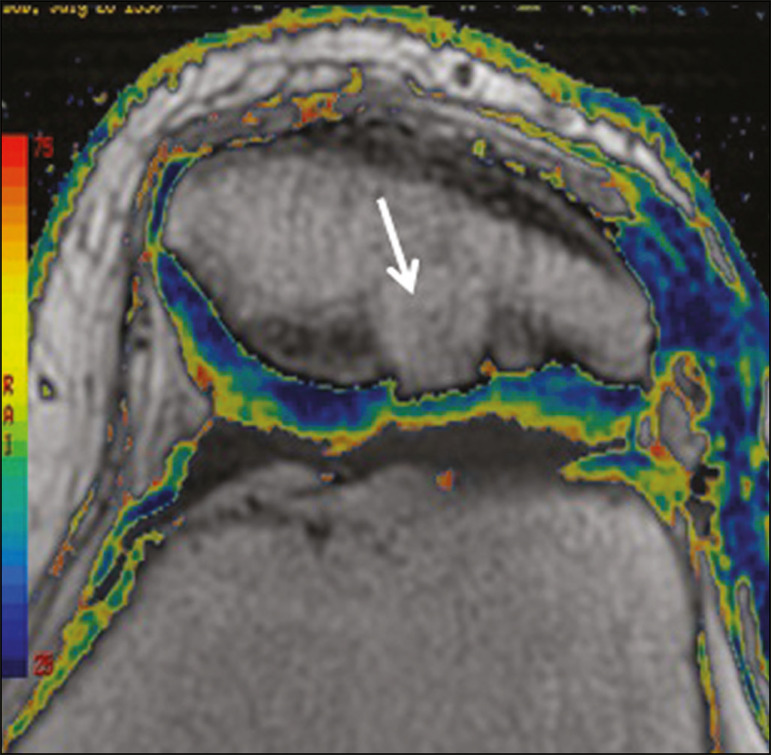
A 39-year-old patient who underwent mosaicplasty with favorable evolution (the same described in Figure 5). T2 mapping shows the stratification of the cartilage surface in the repair zone (arrow), similar to the normal hyaline cartilage in the adjacent aspect of the patella.

Figure 11 shows the stratification of cartilage in a patient without changes. Figures 12 and 13 show cases of patients who underwent surgical manipulation.

### Other techniques

Other compositional techniques have been described in the literature^(10,11)^. Such techniques include measurement of the T1 (spin-lock) relaxation time, late gadolinium-enhanced MRI of cartilage, glycosaminoglycan chemical exchange saturation transfer imaging, sodium imaging, diffusion-weighted imaging, and ultrashort TE imaging.

## CONCLUSION

Radiologists should be familiar with the main techniques for cartilage repair, which were demonstrated in the present study through an illustrative review of didactic cases of surgical techniques, as well as with the postoperative imaging evaluation of such repairs, which we also demonstrated, describing the MRI sequences and the imaging aspects of each case obtained by means of conventional examinations and complementary diagnostic imaging techniques (T2 mapping).

## References

[1] Guermazi A, Roemer FW, Alizai H (2015). State of the art: MR imaging after knee cartilage repair surgery. Radiology.

[2] Blackman AJ, Smith MV, Flanigan DC (2013). Correlation between magnetic resonance imaging and clinical outcomes after cartilage repair surgery in the knee: a systematic review and meta-analysis. Am J Sports Med.

[3] Alparslan B, Ozkan I, Acar U (2007). The microfracture technique in the treatment of full-thickness chondral lesions of the knee. Acta Orthop Traumatol Turc.

[4] Chahal J, Gross AE, Gross C (2013). Outcomes of osteochondral allograft transplantation in the knee. Arthroscopy.

[5] Oztürk A, Ozdemir MR, Ozkan Y (2006). Osteochondral autografting (mosaicplasty) in grade IV cartilage defects in the knee joint: 2- to 7-year results. Int Orthop.

[6] Montoya F, Martínez F, García-Robles M (2013). Clinical and experimental approaches to knee cartilage lesion repair and mesenchymal stem cell chondrocyte differentiation. Biol Res.

[7] Outerbridge RE (1961). The etiology of chondromalacia patellae. J Bone Joint Surg Br.

[8] Outerbridge RE (1964). Further studies on the etiology of chondromalacia patellae. J Bone Joint Surg Br.

[9] MacKay JW, Low SBL, Smith TO (2018). Systematic review and meta-analysis of the reliability and discriminative validity of cartilage compositional MRI in knee osteoarthritis. Osteoarthritis Cartilage.

[10] Guermazi A, Alizai H, Crema MD (2015). Compositional MRI techniques for evaluation of cartilage degeneration in osteoarthritis. Osteoarthritis Cartilage.

[11] Hayashi D, Li X, Murakami AM (2018). Understanding magnetic resonance imaging of knee cartilage repair: a focus on clinical relevance. Cartilage.

[12] Gold GE, Chen CA, Koo S (2009). Recent advances in MRI of articular cartilage. AJR Am J Roentgenol.

[13] Dwyer T, Martin CR, Kendra R (2017). Reliability and validity of the arthroscopic International Cartilage Repair Society classification system: correlation with histological assessment of depth. Arthroscopy.

